# Oral immune priming with *Bacillus thuringiensis* induces a shift in the gene expression of *Tribolium castaneum* larvae

**DOI:** 10.1186/s12864-017-3705-7

**Published:** 2017-04-26

**Authors:** Jenny M. Greenwood, Barbara Milutinović, Robert Peuß, Sarah Behrens, Daniela Esser, Philip Rosenstiel, Hinrich Schulenburg, Joachim Kurtz

**Affiliations:** 1 Institute for Evolution and Biodiversity, University of Münster, Hüfferstrasse 1, 48149 Münster, Germany; 2 Institute of Science and Technology Austria, Am Campus 1, 3400 Klosterneuburg, Austria; 3 Current Address: Stowers Institute for Medical Research, 1000 East 50th Street, Kansas City, MO 64110 USA; 4 Institute of Clinical Molecular Biology, Christian-Albrechts University Kiel, Schittenhelmstr. 12, 24105 Kiel, Germany; 5 Zoological Institute, Christian-Albrechts University Kiel, Am Botanischen Garten 1-9, 24118 Kiel, Germany

**Keywords:** RNA-sequencing, Immune priming, *Tribolium castaneum*, Host-parasite interaction, *Bacillus thuringiensis*

## Abstract

**Background:**

The phenomenon of immune priming, i.e. enhanced protection following a secondary exposure to a pathogen, has now been demonstrated in a wide range of invertebrate species. Despite accumulating phenotypic evidence, knowledge of its mechanistic underpinnings is currently very limited. Here we used the system of the red flour beetle, *Tribolium castaneum* and the insect pathogen *Bacillus thuringiensis* (*Bt*) to further our molecular understanding of the oral immune priming phenomenon. We addressed how ingestion of bacterial cues (derived from spore supernatants) of an orally pathogenic and non-pathogenic *Bt* strain affects gene expression upon later challenge exposure, using a whole-transcriptome sequencing approach.

**Results:**

Whereas gene expression of individuals primed with the orally non-pathogenic strain showed minor changes to controls, we found that priming with the pathogenic strain induced regulation of a large set of distinct genes, many of which are known immune candidates. Intriguingly, the immune repertoire activated upon priming and subsequent challenge qualitatively differed from the one mounted upon infection with *Bt* without previous priming. Moreover, a large subset of priming-specific genes showed an inverse regulation compared to their regulation upon challenge only.

**Conclusions:**

Our data demonstrate that gene expression upon infection is strongly affected by previous immune priming. We hypothesise that this shift in gene expression indicates activation of a more targeted and efficient response towards a previously encountered pathogen, in anticipation of potential secondary encounter.

**Electronic supplementary material:**

The online version of this article (doi:10.1186/s12864-017-3705-7) contains supplementary material, which is available to authorized users.

## Background

Evolution is a dynamic process and nowhere is this better exemplified than in host-parasite interactions. Hosts must perpetually mount defences in order to ameliorate the damage done by parasites, whilst the parasites themselves must evolve to avoid or temper these defences [1–3]. Such resistance or virulence is achieved by Darwinian processes through selection over several host and parasite generations. However, adaptation to parasites can occur within the individual’s lifetime through adaptive immunity, acquired resistance resulting from primary contact that grants survival benefits upon secondary encounter. Acquired immunity was originally thought to be restricted to vertebrates, while invertebrates were supposed to only possess innate immunity. However, evidence from phenotypic analyses have shown that invertebrates may also have some level of immune memory that is often denoted as ‘immune priming’ [4–8]. A particularly compelling aspect emerging from such studies is that the host response is sometimes specific to the pathogenic agent [6, 9, 10]. Such observations have led to suggestions that the boundary between innate and acquired immunity is blurred [11]. Priming in insects can be achieved by haemocoelic infection (pricking) with bacterial components, inactivated or low-dose pathogens [5, 9, 12] and by oral consumption of live bacteria or bacteria-derived components [13–15]. Despite accumulating evidence for immune priming in insects, knowledge of its mechanistic underpinnings is currently limited (for review see, [16]). Insects possess no known comparable system to vertebrates in terms of an underlying genetic basis for acquired immunity, although some candidates have been proposed, e.g. Dscam [17]. Previously, strategies for finding resistance genes involved a time- and knowledge-intensive candidate gene approach, but with the recent advances in sequencing technologies it has become tractable to efficiently explore insect immunity on a genome-wide basis [18, 19]. To date, such genomic approaches have rarely been applied to explore the mechanistic basis of immune priming [20, 21]. Here, we used the red flour beetle, *Tribolium castaneum* and the insect pathogen *Bacillus thuringiensis* to explore the genetic underpinnings of oral immune priming [15]. *T. castaneum* is a major pest of food grain [22]. This species has become a powerful model organism also for studies of insect immunity, with a fully-sequenced genome [23] and more recently, established protocols for studying host-parasite interactions using the pathogen *B. thuringiensis* [9, 24, 25]. It has previously been shown that *T. castaneum* has enhanced survival to *B. thuringiensis* infection after prior exposure to heat-killed bacteria by pricking infection [9] and after exposure to spore supernatant via the oral route [15]. Gene expression strongly differs for infection with live bacteria for these routes [19]. To further our understanding of insect acquired immunity, we here focussed on the oral route of infection and assessed how the priming treatment affects gene expression upon later challenge exposure. For priming, we used filter-sterilised spore supernatants that do not contain any live bacteria or spores to exclude potentially confounding effects of infection upon priming. In addition, we compared priming responses to two strains of *B. thuringiensis*; one of these strains is orally pathogenic to *T. castaneum* and has previously been identified as an effective priming agent, while the other one is neither pathogenic to *T. castaneum*, nor does priming with it lead to enhanced survival [15]. We compared transcriptomes of primed and non-primed larvae upon challenge and identified a priming-specific pattern of gene expression that was clearly distinct from the infection response. Further analysis revealed a number of candidate genes, which provide a new basis to study the molecular underpinnings of immune priming in insects.

## Results

### *Priming with* Btt *elicits a differential gene expression response in* T. castaneum

**Fig. 1:**
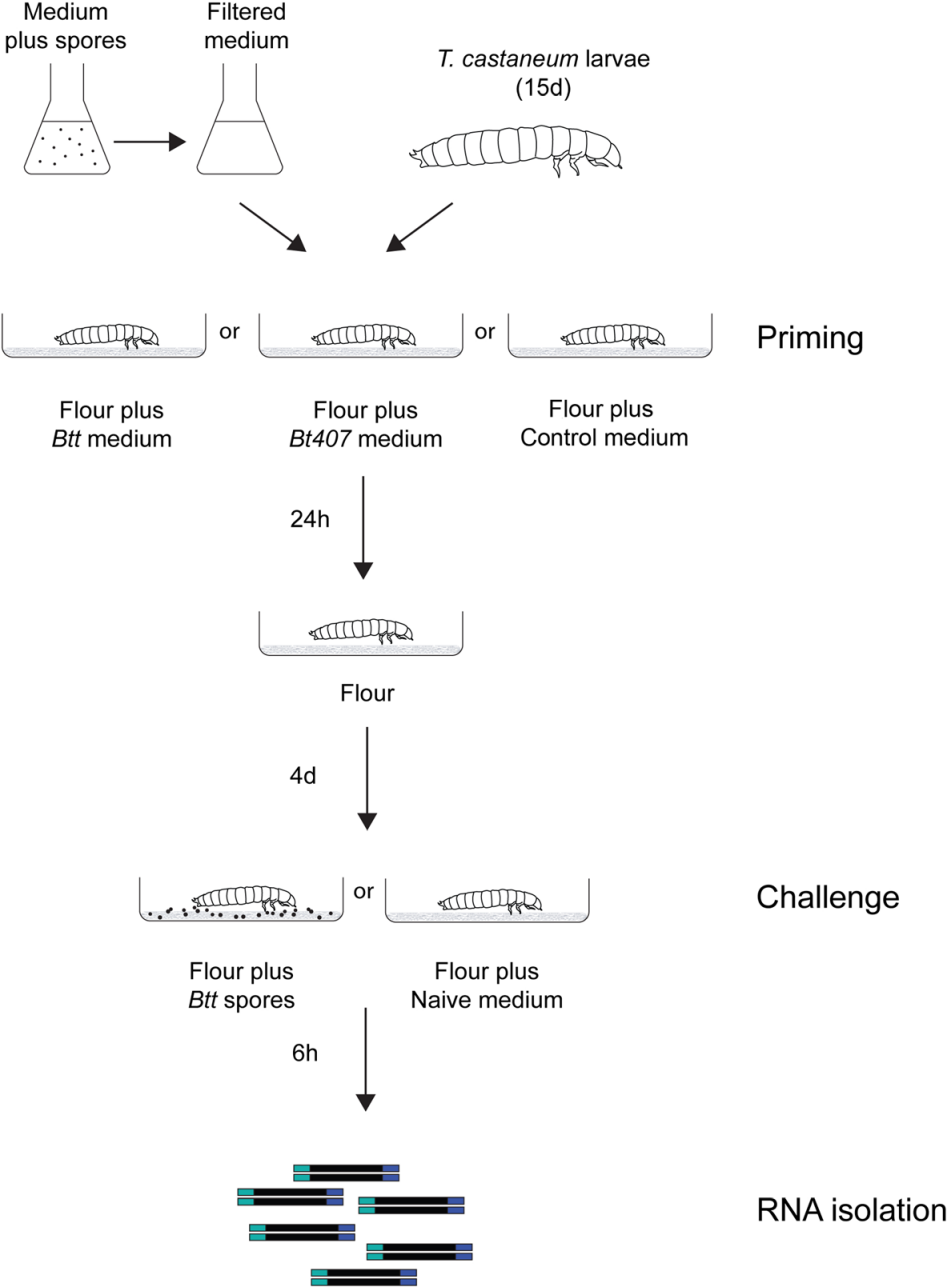
Experimental design. To induce priming, larvae (15 days after oviposition) were exposed to spore-culture supernatants and a medium control (unconditioned medium) for 24 h. Subsequently, larvae were transferred to naïve diet (flour + PBS) for 4 days and exposed to a lethal concentration of spores or naïve diet (flour + PBS). After 6 h, pools of 32 larvae were sampled in triplicates from each group (6 groups in total) and their RNA was isolated for library preparation

We performed RNA-seq experiments to identify the molecular basis of oral immune priming in *T. castaneum* larvae [15]. For this, larvae were orally primed with spore-culture supernatants of either the priming-inducing *Btt* strain, or the *Bt407* ^*-*^ strain, which does not confer survival benefits upon subsequent infection with *Btt*. Both groups (‘*Btt*’ and ‘*Bt407* ^*-*^’ priming) and an additional medium-control group (‘control’ priming) were subsequently orally challenged with *Btt* spores or were left naïve, resulting in six treatment groups, all in triplicates, i.e. 18 sequenced libraries (see Methods and Fig. 1). On average, 51.6 million raw reads were generated per sample and 46 million reads per sample remained after filtering, of which 78% could be mapped to the *T. castaneum* genome. 12288 of the 12777 annotated genes were detected. We found a total of 1610 genes up-regulated and 1448 down-regulated compared to control treatment samples (i.e. control priming with medium and left naïve for challenge). All differentially expressed genes in the different priming-challenge treatments (compared to fully naive control) are listed in Additional file 1: Table S1.

**Fig. 2:**
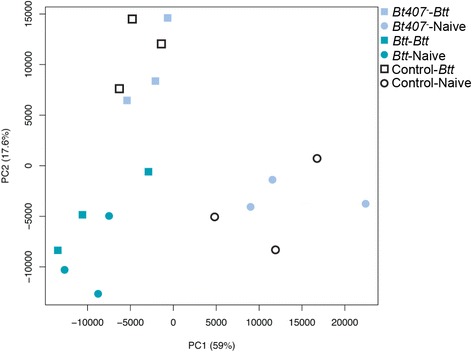
Principle component analysis for all samples 6 h after exposure to *Btt*-contaminated or naïve diet. The plot shows clustering of samples according to treatment group and replicate, based on FPKM expression values and the percentage of variance described by the first two principle components

A principle components analysis (PCA, Fig. 2) showed that the primary axis (describing 59% of the variance) distinguishes among those samples exposed to *Btt* at any stage in the experiment (priming or challenge, i.e. *Btt*-*Btt*, *Btt*-Naive, Control-*Btt*, *Bt407* ^-^-*Btt*) and those having no contact with *Btt* at any stage in the protocol (Control-Naive and *Bt407* ^-^-Naive) and therefore separates beetles by expression response to pathogenicity. The secondary axis (17.6%) distinguishes between beetles that were not primed but were challenged with *Btt* (*Bt407* ^-^-*Btt* and Control-*Btt*), and beetles that were not challenged with *Btt* (*Bt407* ^-^-Naive, Control-Naive and *Btt*-Naive). *Btt*-*Btt* treated beetles cluster with the latter group, rather than those that were not primed but were challenged with *Btt*. This suggests a profound influence of priming with *Btt* spore supernatants on beetles challenged with *Btt* spores.

### *Oral priming with* Btt *spore supernatant prepares larvae for subsequent challenge*

**Fig. 3:**
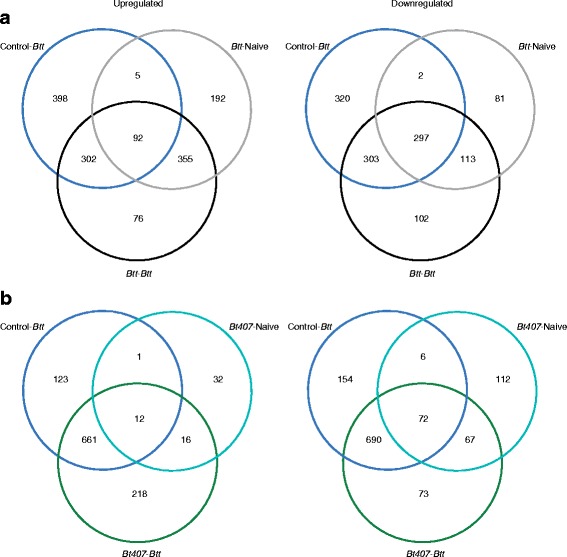
Number of differentially expressed genes 6 h after exposure to *Btt*-contaminated or naïve diet. Venn diagrams representing the number of differentially expressed genes in each treatment group compared to fully naïve control (Control-Naïve). Larvae for the expression analysis were sampled 6 h after challenge with *Btt* or without challenge. **a**. Sets of significantly upregulated and downregulated genes in *Btt*-primed larvae, **b**. Sets of significantly upregulated and downregulated genes in *Bt407* ^*-*^ primed larvae

Consistent with the PCA, Venn diagrams of differentially expressed genes showed many shared changes in expression for different treatments compared with naive beetles (Fig. 3, Additional file 2: Figure S1). For example, 292 genes were up-regulated exclusively among larvae challenged with *Btt* (Control-*Btt*, *Bt407* ^*-*^-*Btt* and *Btt*-*Btt*) and 307 were shared exclusively among *Btt*-challenged larvae that had not been primed or were primed with *Bt407* ^*-*^ (Control-*Btt* and *Bt407* ^*-*^-*Btt*), confirming a strong transcriptional response to *Btt* spore ingestion [19]. Interestingly, 315 genes were specifically up-regulated only in *Btt*-primed larvae (*Btt*-Naive, *Btt*-*Btt*), showing that *Btt*-priming drastically altered gene expression patterns. Large differences in gene regulation were generated in larvae primed by the two *Bt* strains. Few genes in total (61) were up-regulated in larvae primed with *Bt407* ^*-*^ (*Bt407* ^*-*^-Naive) compared with control (Control-Naïve) treatment, consistent with phenotypic data that shows that *Bt407* ^*-*^ confers no priming advantage [15]. However, 112 genes were exclusively down-regulated in *Bt407* ^*-*^-Naive larvae, suggesting that larvae do respond to *Bt407* ^*-*^ cues in their diet, but that any changes in gene regulation do not confer protection upon challenge. Furthermore, since large numbers of differentially expressed genes were exclusively shared in both *Btt*-primed groups (*Btt*-Naive, *Btt*-*Btt*), our results indicate that crucial changes leading to the priming effect already occur before the challenge (during the 4 days between priming and RNA sampling, Fig. 1), strongly influencing gene expression pattern upon challenge itself (*Btt*-*Btt*).

### Immune pathways

**Fig. 4:**
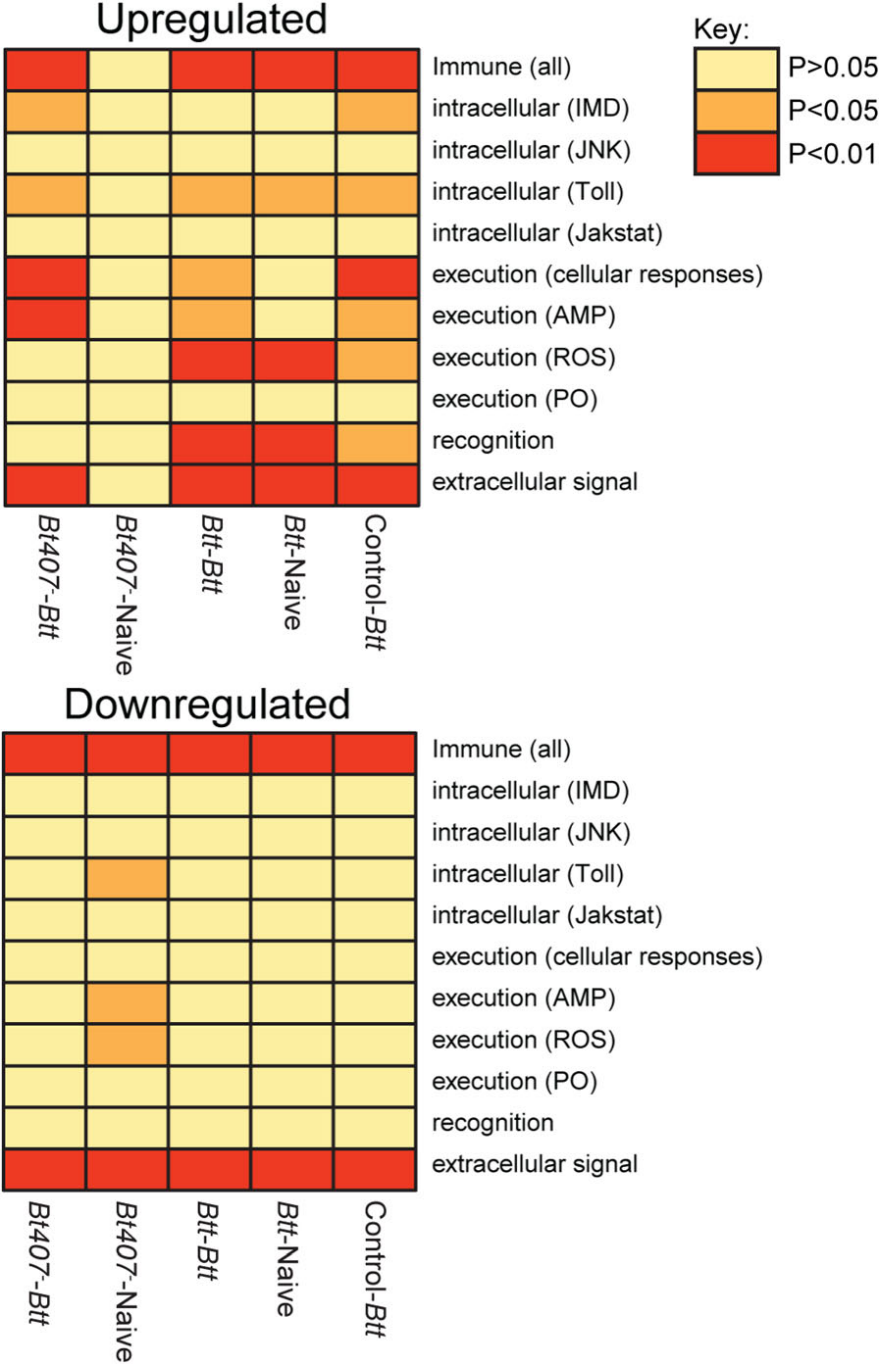
*P*-value heatmaps for different immunity-related categories. *P*-values are based on a Fisher’s exact test of defined immune gene categories [26] of significantly upregulated and downregulated genes for each treatment group compared to the fully naïve control beetles (Control-Naïve)

**Fig. 5:**
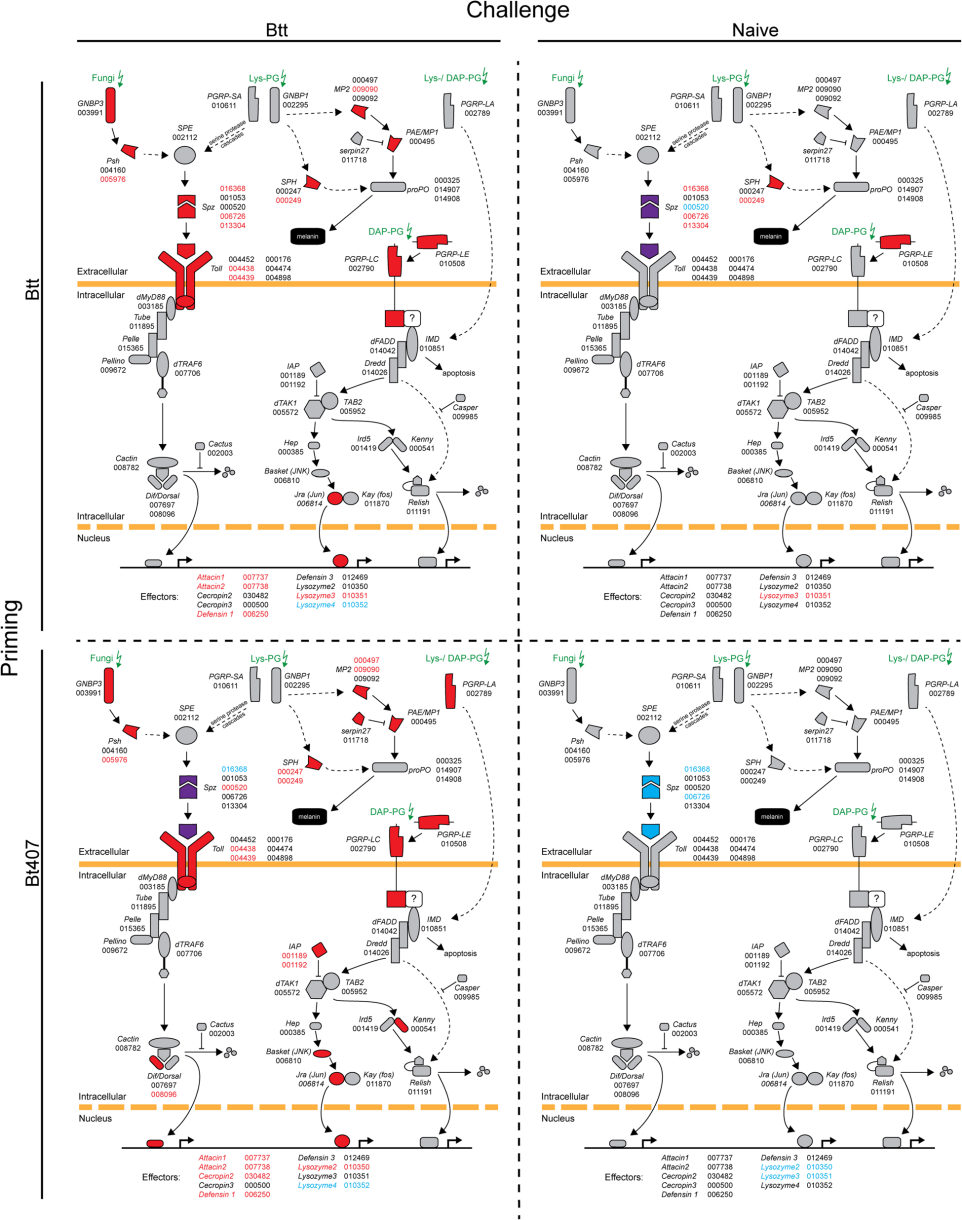
Regulation of the Toll and IMD pathway in relation to priming and challenge treatments. Illustrated are the Toll and IMD pathways after Zou et al., [26] for the two priming treatments, 6 h after the challenge with *Btt* spore-contaminated or naïve diet (flour + PBS). Red color indicates significant upregulation, blue significant downregulation of the respective genes and purple both, significant up- and downregulated genes in the case when genes from the same gene family were reversely expressed. The corresponding official gene IDs (‘TC######’) are specified next to the genes. For effectors, only those that are differentially expressed 6 h after the challenge treatment (*Btt* or naïve) in at least one treatment are indicated with their respective official gene IDs

To further analyse the effect of priming and challenge on immune gene expression, we tested whether certain categories of immune genes identified in *T. castaneum* by Zou et al. [26] showed an enrichment for up- or down-regulated genes (Fig. 4). To investigate in detail the potential role of the Toll and IMD pathways, we also focussed on key components of these pathways and compared their expression between the treatments, as described in Behrens et al. [19] (Fig. 5).

We found a strong signal of differential expression of immune genes (Fig. 4), such that immune genes were overrepresented among both up- and down-regulated genes in all treatment groups, except for *Bt407* ^*-*^-primed and unchallenged beetles, which also did not show any significant enrichment for up-regulated genes in any of the immune subcategory. All other treatment groups showed upregulation of Toll pathway components and extracellular signalling categories. Antimicrobial peptide genes (AMP) genes and genes involved in cellular responses were only up-regulated upon *Btt* challenge, regardless of priming condition. In contrast, recognition genes and reactive oxygen species (ROS) genes were already up-regulated upon priming with *Btt* (but not *Bt407* ^*-*^), even when this was not followed by infection, suggesting that priming leads to an increased ‘alertness’ by up-regulation of immune recognition genes and an elevated level of reactive oxygen based defences. It is further noteworthy that IMD genes that were up-regulated upon infection without priming (Control-*Btt* and *Bt407* ^*-*^ *-Btt*) did not show differential regulation when there was previous priming with *Btt.* Finally, in the *Bt407* ^*-*^-Naive treatment groups, the ROS, AMP and Toll pathways were significantly down-regulated compared with control-primed beetles.

Upon closer inspection of the Toll and IMD pathways, we found many differences for the *Btt* challenged larvae in relation to whether they had been primed with *Btt* or *Bt407* ^*-*^ (Fig. 5). In detail, several genes of the classical immune pathways (e.g. *PGRP-LA*, *Dif*, *Basket*, *IAP*, *Kenny*) were up-regulated upon challenge following inefficient priming (with *Bt407* ^*-*^), suggesting an overall activation of these pathways. In contrast, we found only a reduced number of key genes of the Toll and IMD pathway to be activated in the *Btt* priming-*Btt* challenge scenario.

### *Genes with known functions against* B. thuringiensis *and other pathogens are up-regulated upon priming with* Btt

**Fig. 6:**
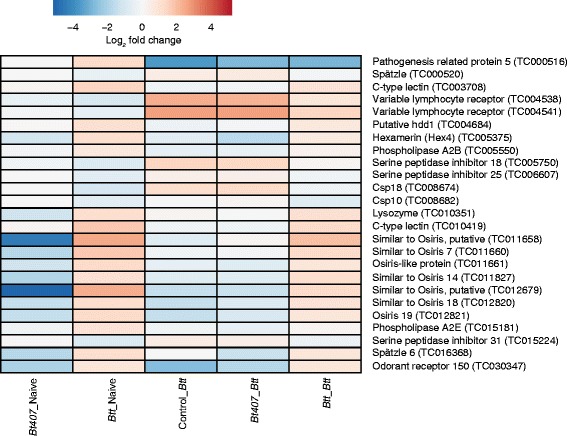
Heatmap of differentially regulated candidate immunity-related genes. Heat maps of a subset of candidate immunity-related genes regulated upon priming or showing reversed expression compared to groups challenged without or after ineffective priming. Heat maps are based on the log2 fold change expression values. Genes are sorted according to increasing TC numbers. Please note that gene descriptions for *T. castaneum* often come from automatic annotations and are not always verified by functional analyses

To narrow down candidates among the genes differentially regulated upon priming (groups *Btt*-Naive and *Btt*-*Btt*; Fig. 3), we screened the literature for known pathogen-related functions of those genes. We found several candidates with a described role in insect immunity to be differentially regulated (Fig. 6, Additional file 3: Figure S2, Additional file 4: Table S2). For example, lysozyme (TC010351) and many of the c-type lectin genes (e.g. TC003708, TC010419) were strongly up-regulated, and have a known function in defence against a variety of bacteria. Lysozymes cut bacterial cell wall components [27–29] and c-type lectins play an important role in pathogen recognition and opsonisation [30–32], and were recently proposed to contribute to specific immune responses, especially in invertebrates [33]. Furthermore, two phospholipase A2 genes (TC015181, TC005550) were found up-regulated upon priming (Fig. 6, Additional file 3: Figure S2). These enzymes participate in the formation of eicosanoids from arachidonic acid [34, 35] and were found to play multiple immune roles in insects, such as in nodulation, prophenoloxidase activation [36, 37] and phagocytosis [34], including responses to bacterial challenge and Toll and Imd pathway activation in *T. castaneum* [35].

It is further noteworthy that we found several genes with a known defence function against orally ingested pathogens and specifically, against *B. thuringiensis*, exclusively in the *Btt* primed larvae (*Btt*-Naïve, *Btt*-*Btt*). Particularly interesting is the hexamerin gene (TC005375), which we found strongly up-regulated compared to control (Fig. 6, Additional file 3: Figure S2). Hexamerin expression and protein concentration were found increased after a bacterial challenge [38–41]. In *Drosophila*, hexamerin was indicated to function as a humoral pro-coagulant, participating in bacteria binding and clot formation [42]. Interestingly, increased hemolymph and gut hexamerin concentrations were found to play a role in the reaction of the cotton bollworm to Cry1Ac toxins produced by a *B. thuringiensis* strain, by aggregating with the toxin crystal and forming an insoluble coagulant [43–45]. A putative Hdd1 defense protein gene (TC013059), associated with gut immune defence and resistance to *B. thuringiensis* toxins was found strongly expressed upon *Btt* priming (Fig. 6, Additional file 3: Figure S2). The expression of this gene was up-regulated after ingestion of bacteria-contaminated diet in the cabbage semilooper [38] and a similar gene was found to be involved in prophenoloxidase activation and nodulation response in the cotton bollworm [46]. Interestingly, a gene of the same class (Hdd11) was found induced in the Cry 1Ab resistant sugarcane borer [47] and in the beet armyworm after ingestion of *B. thuringiensis* VIP toxins [48], indicating its importance in defence against *B. thuringiensis*.

### Immune priming differs from challenge

**Fig. 7:**
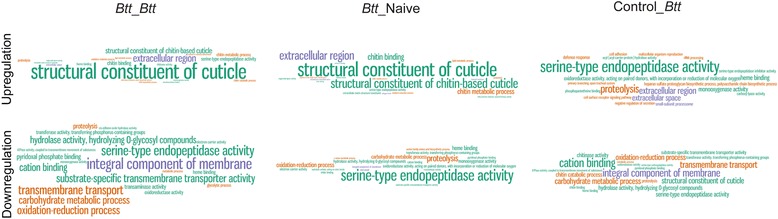
Word clouds of overrepresented GO terms in sets of differentially expressed genes. The R package TopGO was used to perform GO enrichment analyses for each set of differentially expressed genes in different treatment groups, using the weight01 GO graph algorithm and a Fishers Exact Test for significance [74]. *P*-values were scaled according to the scaling factor -log_10_(*p*-value) and the top 20 terms were visualised in Wordle™

We found that 9% (78 of the total 825) of the genes upregulated upon challenge with *Btt* after previous priming (*Btt*-*Btt*) were in fact downregulated in beetles challenged without priming (Control-*Btt)*. The different signature of priming *vs.* challenge was seen in the overrepresentation of gene ontology (GO) terms in the different treatments (Fig. 7, Additional file 5: Table S3). “Structural constituent of cuticle” was the most strongly overrepresented term among up-regulated genes when the larvae were primed with *Btt* (*Btt*-Naive and *Btt*-*Btt*), whereas in larvae only challenged with *Btt* (Control-*Btt*), “serine-type endopeptidase activity” was the most significantly up-regulated term. Interestingly, this GO term was most significantly down-regulated in larvae only primed with *Btt* (*Btt*-Naïve), suggesting an inverse pattern of gene regulation in primed, compared to challenged-only larvae.

When we looked at only *Btt* primed individuals that were not challenged with live spores (*Btt*-Naïve), we found several immune candidates downregulated in this treatment, but upregulated upon challenge-only (Fig. 6, Additional file 3: Figure S2). These were the two genes annotated as variable lymphocyte receptors (TC004538, TC004541), a chemosensory protein Csp 18 (TC008674) as well as two serine peptidase inhibitors (TC005750, TC015224) and a spätzle (TC000520). Conversely, a pathogenesis related protein (TC000516), odorant receptor 150 (TC030347), and several Osiris genes (TC012679, TC011827, TC011661, TC011660, TC011658, TC012820, TC012821) were found up-regulated upon priming, but down-regulated upon challenge (Fig. 6, Additional file 3: Figure S2). Even though different time-points after priming (4 days) and challenge (6h) are here considered, these observations, together with the data shown above, strongly indicate that immune priming differs from challenge.

## Discussion

In this study, we aimed at exploring the molecular underpinnings of oral immune priming in *T. castaneum* larvae [15] using a whole-animal transcriptome sequencing approach. We analysed host gene expression profiles after exposure to bacterial cues (culture supernatants) of two different strains; an orally pathogenic strain (*Btt*) inducing a priming response and conferring survival benefits upon challenge, and a non-pathogenic strain (*Bt407* ^*-*^) where this effect remains absent. Consistent with phenotypic results [15], we found strong differences in host gene expression profiles between the two bacterial strains. Whereas priming with *Bt407* ^*-*^ showed only minor differences compared to the control-priming group in terms of up-regulated genes, priming with *Btt* induced a large set of differentially regulated genes (Figs. 3 and 6, Additional file 3: Figure S2), indicating strong responses of the host to the *Btt* priming diet. Note that priming in our study is achieved by using sterilised spore culture supernatants, such that observed gene expression changes cannot be attributed to an active infection in the host.

We expected two hypothetical scenarios by which immune priming could take place [16, 49]. In the first, priming could induce a sustained immune response that would prophylactically confer a survival benefit upon secondary challenge with live *Btt* [5, 50]. Indeed, we found support for this scenario in our dataset, i.e. gene expression changes upon priming that remained active until the challenge. For example, several genes of the Toll and IMD pathways were found activated prior challenge (i.e. also in the *Btt*_Naive group; Fig. 5, right upper panel), which could represent an anticipatory response based on potentially higher amounts of antimicrobial peptides that are ‘ready-to-use’ upon challenge. This hypothesis is also based on the fact that the Toll and IMD pathways are in a somewhat ‘active state’ (i.e. upregulation of upstream factors such as *PGRP-LE* for IMD and *Spätzle* for Toll) after priming with *Btt*, when not followed by challenge. Second, priming could lead to an anticipatory immune defence that is recalled upon challenge, such that a stronger and/or faster response could take place, facilitating the rapid elimination of pathogens. Such priming would rely on immune memory in a more narrow sense, i.e., the ability of an immune system to store and recall the information on a previously encountered pathogen [7, 8, 51]. In both of the above mentioned cases (sustained or recalled), the type of response upon priming and challenge would be qualitatively similar. Such a scenario was recently observed in trans-generational immune priming in bumble bees, where worker offspring coming from primed queens exhibited overlapping expression signatures with workers that were directly exposed, but whose mothers were not primed [20].

By contrast, the activated immune repertoire that we here observed upon priming seems to a certain degree to be qualitatively different from the one mounted upon infection without a previous priming. Intriguingly, a similar observation of a qualitatively distinct priming response was recently reported for immune priming in the snail *Biomphalaria glabrata* with the trematode parasite *Schistosoma mansoni* [21, 52]. In this system, priming resulted in a shift from cellular to humoral immune reactions. When comparing groups that were challenged with or without preceding priming in our study, we found a large set of genes regulated in the *Btt* primed-*Btt* challenged group, many of which are known immune candidates. Since the majority of those genes were already regulated in the group receiving no challenge (*Btt*-Naïve), this suggests that the observed survival benefit in *T. castaneum* larvae results from the induction of a specific set of genes, early after the exposure to bacterial cues in their environment, and that such induction results in largely different gene expression than the one activated upon challenge without the previous priming. It would be interesting to see how prevalent these scenarios are among invertebrate phyla, i.e., whether the priming gene expression is different or similar compared to a secondary challenge.

Compared to *Drosophila*, *T. castaneum* seems to mount a rather promiscuous immune response after bacterial challenge, by concomitantly upregulating Toll and IMD pathways in response to bacteria (Gram^-^ or Gram^+^) [53, 54]. Indeed, challenge without the previous priming induced an overall regulation of the Toll and IMD pathways in our study as well (Figs. 4 and 5, left lower panel); however, only a reduced number of those genes were upregulated upon challenge when there was previous priming (Figs. 4 and 5, left upper panel). We even found many of the priming-specific genes to show an inverse regulation compared to their regulation upon challenge without the priming, a pattern that was also visible in the different GO term overrepresentation results (Fig. 7). We hypothesise that this “shift” in gene expression indicates activation of a more targeted and more efficient response following information on previously encountered pathogens, in anticipation of a potential secondary encounter. For example, priming induced several novel effector genes, not normally expressed upon infection, like hexamerin, pathogenesis related protein 5, lysozyme and hdd1 defence protein, all with a reported role in defence against orally ingested pathogens and *Bacillus thuringiensis*. Activation of a narrower, more suitable response type towards a specific pathogen would certainly prove beneficial when an infection is expected.

It is difficult to narrow down the oral priming response to only a few candidate genes. Rather, priming with *Btt* seems to mount an orchestrated response of various signalling and effector molecules, together contributing to survival benefits upon secondary exposure to lethal concentration of spores. Of note is however, that in order to understand phenotypic data, we looked at gene regulation 4 days after priming had occurred. Although this still had a strong influence on gene expression upon challenge (*Btt*_*Btt* treatment group, Figs. 3 and 6, Additional file 3: Figure S2), it may be that looking at earlier timepoints would reveal important candidates more clearly (e.g., by stronger regulation). Hexamerin though, could play a particularly important role. Hexamerins, or similar lipoproteins were found to coagulate and bind *Bt* toxins, rendering them inactive [44, 45]. Strong hexamerin regulation may suggest formation of a trap by crosslinking with other hexamerin molecules, thus facilitating binding of the toxins and/or bacteria and inactivating them [44, 45]. Hexamerins may therefore serve as a first line of defence in the gut [45], especially if accumulating in the gut upon priming, trapping the toxins and bacteria and preventing them from reaching the midgut epithelium.

Of note here is that the information on gene identity in *T. castaneum* often comes from automatic annotations based on sequence similarities and while some genes are well annotated (e.g., hexamerin, lysozyme), others (e.g., variable lymphocyte receptors, Fig. 6) are not verified by functional analyses. Similarly, we found many genes strongly regulated for which no annotation so far exists or with no described function in other insects, as is the case for the many Osiris-like genes with characteristic expression patterns (Fig. 6). The Osiris gene family is well-conserved across insects, but its function is so far largely unknown [55, 56].

It is not clear which bacteria-derived components induce priming in our system, as the supernatant of a sporulating culture may be abundant in various candidate molecules or pathogen-associated molecular patterns; remnants of the cell wall components remaining after the cell lysis or secreted non-proteinaceous components or toxins that remained in the supernatant after filter-sterilisation. Such bacterial cues might damage the host tissue and serve as a danger signal, activating host immunity [57], which could be absent in the non-pathogenic *Bt407* ^*-*^ strain. In this context, it is noteworthy that *Bt407* ^-^, in contrast to *Btt*, does not have the beetle-specific Cry toxin that breaks the gut barrier. However, upon preparation of the priming diet, bacterial culture is centrifuged such that no crystal toxins should remain in the supernatnats, except potentially in a form of loose monomers, but this needs to be investigated. Alternatively, certain molecules might be affecting the resident microbiota, further promoting a primed response. Indeed, a recent study showed that, similar to the mosquito-*Plasmodium* system [14, 58], microbiota play a crucial role in the oral priming in our system as well [59], but the mechanisms for this dependence and how they relate to the specific gene expression patterns found here are still unknown. Interestingly, we found *PGRP-LE* activated after priming with *Btt* (*Btt*_Naive, Fig. 5, right upper panel), which is a key gut bacterial sensor in *Drosophila* involved in balanced responses to pathogenic bacteria and microbiota [60]. Further research is needed to see whether and how this interesting gene is involved in microbiota regulation of priming in our system.

Regarding the question of specificity of priming responses, it is interesting that the ‘non-efficient’ *Bt407* ^*-*^ priming was not without any effects. Compared to fully naive controls, we found a quite clear pattern of down-regulation of genes (Fig. 3), in particular for Toll, AMP and ROS-mediated defences (Fig. 4), which might also be the cause for the observed absence of enrichment for up-regulated immune recognition upon *Btt* challenge (Fig. 4). However, the reasons for these effects are currently unclear and need further study.

A puzzling question is, why a potentially more effective immune response would be activated only when priming occurred, but not constitutively or upon challenge without priming? First, as with other immune defence, priming with *Btt* seems to be costly; primed larvae grow and develop more slowly than the controls [15]. It thus may depend on the epidemiology as to whether it is worth paying these costs. Second, in periods of high bacterial load in a population of beetles, priming might actually regularly occur, e.g. via cannibalising infected larvae [61]. Priming-eliciting cues could be present in cadavers as the result of bacterial sporulation such that eating infected cadavers could prove beneficial for survival. However, little is currently known about *Bt* epidemiology and how host and pathogen interact in nature; these topics require further research.

## Conclusions

We here show that oral priming with spore culture supernatants of *B. thuringiensis tenebrionis* is achieved by extensive transcriptome changes in *T. castaneum* that are specific to priming with *Btt*, but absent from priming with the non-infectious strain *Bt407* ^*-*^. A unique pattern of gene expression was found that is different from challenge without the previous priming. Such a shift in the expression pattern towards a potentially more effective response is very intriguing and it would be interesting to test if this phenomenon is bacteria-specific and whether such a response type could also be found in other invertebrates that show immune priming.

## Methods

### Insects

For all experiments we used the wild type strain of *Tribolium castaneum*, Croatia 1 (Cro1), which was collected in May 2010 in Croatia [24]. This strain was adapted to lab conditions for more than 20 generations (~18 months). Beetles were reared on flour (type 550) with 5% brewer’s yeast at 30°C with a 12/12 h light/dark cycle and 60% relative humidity.

### Bacteria and spore cultivation

*Bacillus thuringiensis* bv*. tenebrionis* (*Btt*) was obtained from the Bacillus Genetic Stock Center (BGSC, Ohio State University, USA) and *Bacillus thuringiensis 407* ^*-*^ (*Bt407* ^*-*^) was kindly provided by Dr. Christina Nielsen-Leroux, Institut National de Recherche Agronomique (La Minière, 78285 Guyancourt Cedex, France). Before using in experiments, bacteria were subcloned five times on LB-Agar and glycerin stocks were stored at -80°C. Spore cultures of *Btt* and *Bt*407^-^ were produced as previously described [24] and centrifuged at 2900 x g at room temperature for 12 min. Spores were washed and subsequently resuspended in phosphate buffered saline (PBS, Calbiochem®), counted using a Thoma counting chamber (0.02mm depth) and used for challenge immediately.

### Experimental design

The priming experiment was done as in Milutinović et al. [15] (see Fig. 1). For this, spore cultures of *Btt* and *Bt*407^-^ were centrifuged and the supernatants subsequently filter-sterilised, first using a 0.45 μm and then a 0.2 μm cellulose acetate filter (Whatman GmbH). Flour with yeast was added to the supernatant (0.15 g/mL of supernatant). Diet for the control larvae was prepared by mixing the flour with sterile sporulation media. Such liquid diet was pipetted into wells of a 96-well plate (Sarstedt, Germany) and dried in the oven at 36°C overnight. The next day, similar-sized *T. castaneum* larvae (15 days after a 24 h oviposition) that were cultivated under standard conditions were individually exposed to the priming or control diet (sterile sporulation media) for 24 h and transferred to a naive diet of flour discs obtained by mixing flour and PBS. Larvae were kept on the naive diet for 4 days after which they were similarly exposed to spore-containing (5 × 10^9^ mL^-1^ *Btt* spores in PBS mixed with flour) or naive diet for 6 h and sampled for the transcriptome analysis. This timepoint was used since our previous study showed that sampling 6 h after the challenge gives a clear expression signature, compared to already weaker expression after 18 h [19]. Hence, the sampled treatments were as follows: *Btt* primed-*Btt* challenged (*Btt*-*Btt*), *Bt407* ^*-*^ primed-*Btt* challenged (*Bt407* ^*-*^-*Btt)*, Control-*Btt* challenged (Control-*Btt*), *Btt* primed-Naïve (*Btt*-Naive), *Bt407* ^*-*^ primed-Naïve (*Bt407* ^*-*^-Naïve), Control-Naïve. Each treatment was replicated 3 times, with a pool of 32 larvae each.

### Sample preparation, library construction and sequencing

For each treatment group, three replicate RNA libraries, each consisting of the 32 pooled *T. castaneum* individuals were produced. Total RNA from frozen beetles was isolated using mirVana^TM^ miRNA Isolation Kit (Ambion) according to the instructions of the manufacturer. The libraries for the whole transcriptome sequencing were created with the Illumina TruSeq RNA Library Prep kit (version February 2012, Part# 15026495 Rev. B). After cluster generation on the cBot with the TruSeq PE Cluster Kit v3, the sequencing was performed with the TruSeq SBS Kit v3 (200 cycles) on two lanes of the Illumina HiSeq 2000.

### Transcriptomic analysis

The transcriptomic assembly and analysis closely followed the procedures described by Behrens et al. [19]. Before mapping, a number of filtering steps were performed on the data. Firstly, Illumina quality-failed reads were removed from the read files, and adaptor sequences were removed using the package SeqPrep [62]. Then Seqtk [63] was used to trim the first 13 base pairs of sequence from the reads to remove biases in nucleotide composition due to random hexamer priming [64], which improved the number of reads mapping to the genome.

After filtering, Tophat v2.0.11 [65] was used to map the reads to the Tribolium 3.0 reference genome downloaded from Beetlebase (Kim et al. [66]). A separate, more recent annotation file, incorporating improved gene models taking advantage of transcriptomic data was downloaded from the iBeetle website [67, 68] and used to guide the mapping process, as well as the subsequent assembly and differential expression analyses.

Next, Cufflinks v.2.2.1 [69] was used to quantify the transcripts against the reference.gtf file, using default parameters. Cuffmerge was used to merge the individual assemblies into a comprehensive transcriptome and the Cuffdiff utility [70] was used to normalise the data using upper-quartile normalisation and to quantify differential expression of genes across samples; a value of *p* < 0.05, FDR < 0.05 was used to identify genes with significant differential expression. Data were imported into R [71] for further statistical analysis.

Principle components analysis (PCA) was used to summarise the distribution of gene expression values of the samples using functions in the R base package and Venn diagrams were generated for the data using the R package VennDiagram [72].

In order to generate functional terms associated with the genes of interest, the software Blast2GO [73] was used to annotate the iBeetle *T. castaneum* genome annotation [67]. The R package TopGO was then used to generate GO enrichments for each of our treatment comparisons of interest, using the weight01 GO graph algorithm and Fishers exact test for significance [74]. Results of the 20 most significant terms were visualised with Wordle™ after the *p*-values were scaled according to -log_10_(*p*-value) [19, 75]. Subsequently, these results were merged with Gene Ontology terms [76] downloaded from Ensembl Biomart [77] and results of a BlastP search [78] using default parameters, to further add gene information for interpretation of the results.

Finally, immunity genes identified by Zou et al. [26] were tested for enrichment in up- or downregulated genes of each treatment using a Fisher’s exact test. *P*-values were normalised using the Benjamini-Hochberg correction method [79]. Zou et al. [26] identified around 300 candidate defense proteins based on sequence similarity to homologs known to participate in immune responses. They further characterized these genes with phylogenetic analyses of immune gene families and RT-qPCR analyses after bacterial and fungal pricking.

## Additional files

Additional file 1: Table S1. Lists of all differentially expressed genes in the different priming-challenge treatments (compared to the fully naïve control; xlsx file). Relevant columns include the following: sample_1 and sample_2 – treatment groups being compared; Normalised FPKM sample_1 and sample_2 – FPKM of samples being compared; log2(fold_change) – log2(FPKM sample 2/FPKM sample 1), i.e. negative means sample 1 upregulated compared with sample 2, positive means sample 2 upregulated compared with sample 1; cuffdiff test_statistic – test statistic of differential expression test; *p*_value – *p*-value of differential expression test; q_value (FDR correction) – adjusted *P*-value of differential expression test. (XLSX 598 kb)

Additional file 2: Figure S1. Number of differentially expressed genes 6 h after exposure to *Btt*-contaminated or naïve diet. Venn diagrams representing the number of differentially expressed genes in each treatment group compared to fully naïve control (Control-Naïve). Larvae for the expression analysis were sampled 6 h after challenge with *Btt* or without challenge. A. Sets of significantly upregulated genes in all treatments, B. Significantly downregulated genes in all treatments. (PDF 1.12 kb)

Additional file 3: Figure S2. Barplots of candidate immunity-related genes. Barplots of a subset of candidate immunity-related genes regulated upon priming or showing reversed expression compared to groups challenged without or after ineffective *Bt407* priming (see also Fig. 6.) Y-axis shows FPKM values. Error bars show 95% confidence intervals. Please note that gene descriptions for *T. castaneum* often come from automatic annotations and are not always verified by functional analyses. (PDF 9.42 kb)

Additional file 4: Table S2. Gene description summary for Additional file 3: Figure S2. The table shows the au2 and TC numbers as well as the gene description for the genes shown in Fig. 6 and Additional file 3: Figure S2. Please note that gene descriptions for *T. castaneum* often come from automatic annotations and are not always verified by functional analyses. (DOCX 69 kb)

Additional file 5: Table S3. Lists of top 20 overrepresented GO terms in Btt exposed treatment groups (compared to fully naïve control). Methodology as described for Fig. 7. The columns are as follows: GO ID – Gene Ontology unique ID of the overrepresented GO term; GO term – text descriptor of the overrepresented GO term; total no.genes annotated – total number of genes in the *T. castaneum* genome which are annotated with this GO term; observed – number of genes over- or underrepresented in the treatment found to be annotated with this GO term; expected – number of genes over- or underrepresented in the treatment expected to be annotated with this GO term under the null hypothesis; *P*-value – *P*-value of the Fisher’s test found using the Weight01 algorithm of [74]; Ontology – GO category to which each GO term belongs (MF – Molecular Function; CC – Cellular Component; BP – Biological Process). (XLSX 21 kb)

## Abbreviations

AMP: Antimicrobial peptide
*Bt407*^*-*^: *Bacillus thuringiensis 407* ^*-*^
*Btt*: *Bacillus thuringiensis* bv*. tenebrionis*
Cro1: Croatia 1 beetle population
Dscam: *Down syndrome cell-adhesion molecule*
GO: Gene ontology
IMD: Immune deficiency pathway
PCA: Principle components analysis
ROS: Reactive oxygen species
