# Supplementary figures and images for: Oral immune priming with Bacillus thuringiensis induces a shift in the gene expression of Tribolium castaneum larvae
Source: BMC Genomics. 2017 Apr 26;18:329. doi: 10.1186/s12864-017-3705-7 (PMC5405463; doi:10.1186/s12864-017-3705-7)

A.

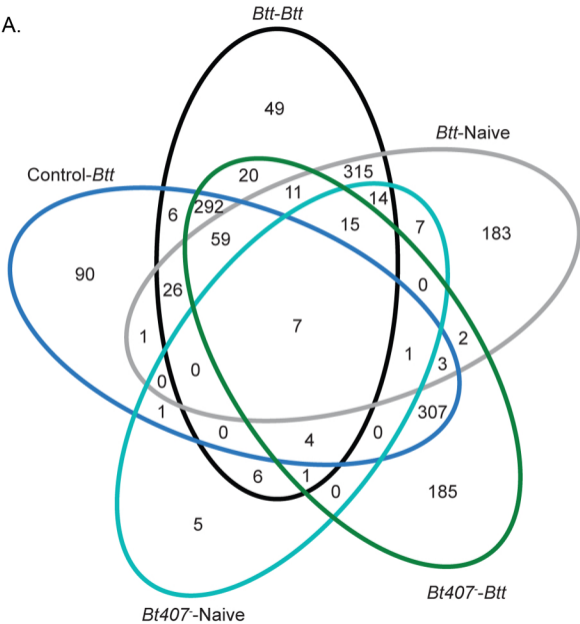

B.

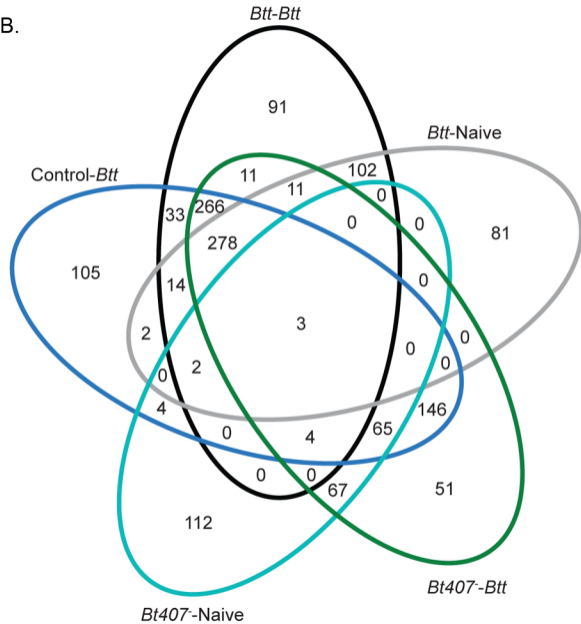

Supplement: Supplementary file 2 — Number of differentially expressed genes 6 h after exposure to Btt-contaminated or naïve diet. Venn diagrams representing the number of differentially expressed genes in each treatment group compared to fully naïve control (Control-Naïve). Larvae for the expression analysis were sampled 6 h after challenge with Btt or without challenge. A. Sets of significantly upregulated genes in all treatments, B. Significantly downregulated genes in all treatments. (PDF 1.12 kb) [file 12864_2017_3705_MOESM2_ESM.pdf]

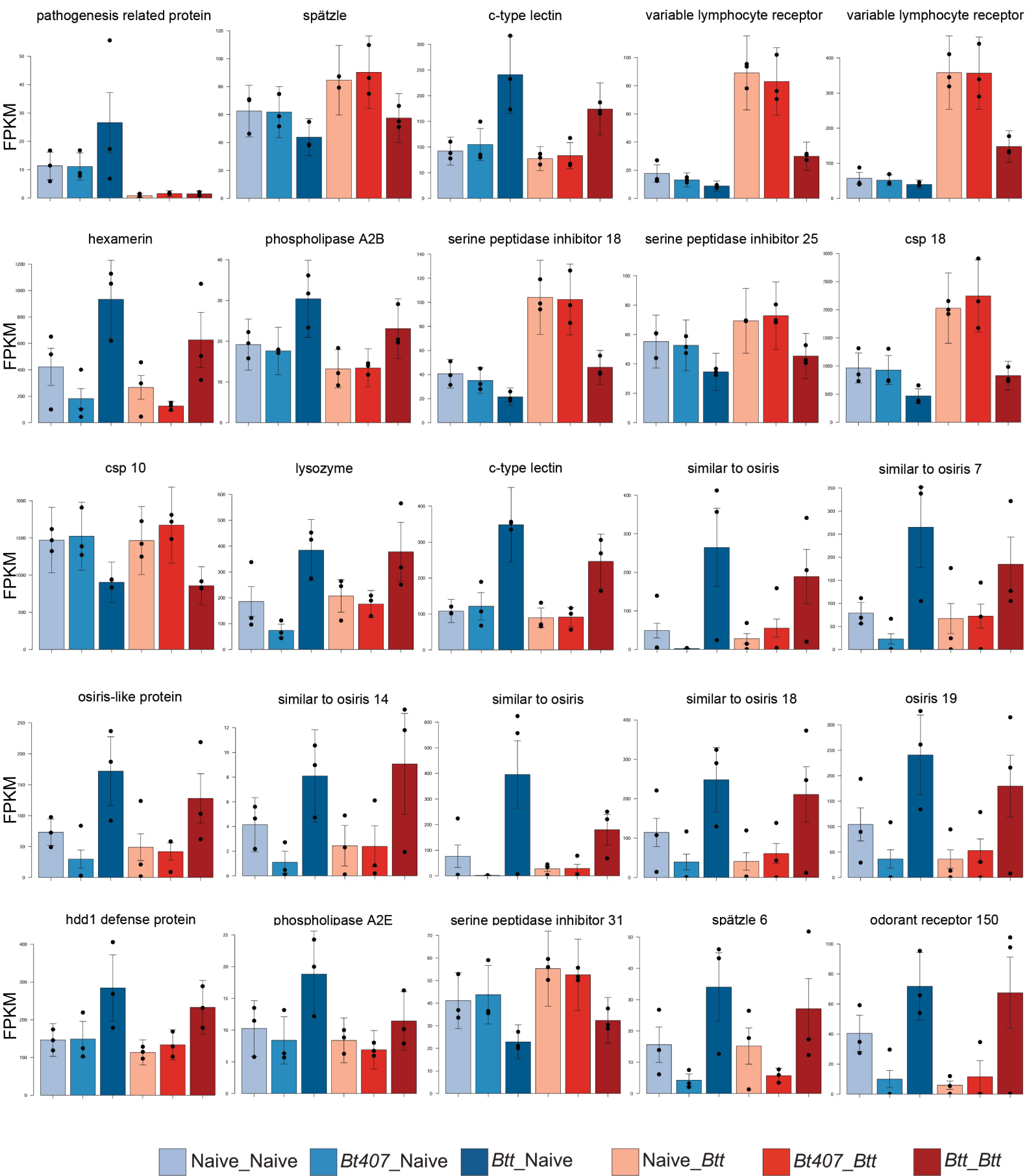

Supplement: Supplementary file 3 — Barplots of candidate immunity-related genes. Barplots of a subset of candidate immunity-related genes regulated upon priming or showing reversed expression compared to groups challenged without or after ineffective Bt407 priming (see also Fig. 6.) Y-axis shows FPKM values. Error bars show 95% confidence intervals. Please note that gene descriptions for T. castaneum often come from automatic annotations and are not always verified by functional analyses. (PDF 9.42 kb) [file 12864_2017_3705_MOESM3_ESM.pdf]
