# Supplementary material for: Oral immune priming with Bacillus thuringiensis induces a shift in the gene expression of Tribolium castaneum larvae
Source: BMC Genomics. 2017 Apr 26;18:329. doi: 10.1186/s12864-017-3705-7 (PMC5405463; doi:10.1186/s12864-017-3705-7)
Supplement: Supplementary file 4 — Gene description summary for Additional file 3: Figure S2. The table shows the au2 and TC numbers as well as the gene description for the genes shown in Fig. 6 and Additional file 3: Figure S2. Please note that gene descriptions for T. castaneum often come from automatic annotations and are not always verified by functional analyses. (DOCX 69 kb) [file 12864_2017_3705_MOESM4_ESM.docx]

**Table S2. Gene description summary for Figure S2**

| **au2/TC ID** | **Gene Description** |
| --- | --- |
| au2.g2861/TC000516 | pathogenesis related protein 5 |
| au2.g2852/TC000520 | Spätzle |
| au2.g4616/TC003708 | c-type lectin |
| au2.g2049/TC004538 | variable lymphocyte receptor |
| au2.g2053/TC004541 | variable lymphocyte receptor |
| au2.g12113/TC005375 | hexamerin (Hex4) |
| au2.g11814/TC005550 | phospholipase A2B |
| au2.g11524/TC005750 | serine peptidase inhibitor 18 |
| au2.g11875/TC006607 | serine peptidase inhibitor 25 |
| au2.g9582/TC008674 | Csp18 |
| au2.g9574/TC008682 | Csp10 |
| au2.g4925/TC010351 | lysozyme |
| au2.g7166/TC010419 | c-type lectin |
| au2.g10816/TC011658 | similar to Osiris, putative |
| au2.g10813/TC011660 | similar to osiris 7 |
| au2.g10812/TC011661 | osiris-like protein |
| au2.g10590/TC011827 | similar to Osiris 14 |
| au2.g10588/TC012679 | similar to Osiris, putative |
| au2.g10832/TC012820 | similar to Osiris 18 |
| au2.g10833/TC012821 | osiris 19 |
| au2.g7974/TC013059 | hdd1 defense protein |
| au2.g275/TC015181 | phospholipase A2E |
| au2.g11623/TC015224 | serine peptidase inhibitor 31 |
| au2.g8894/TC016368 | Spätzle 6 |
| au2.g6856/TC030347 | odorant receptor 150 |
